# Dynamic Coherent Diffractive Imaging with Modulus Enforced Probe and Low Spatial Frequency Constraints

**DOI:** 10.3390/s25072323

**Published:** 2025-04-06

**Authors:** Yingling Zhang, Zijian Xu, Bo Zhao, Xiangzhi Zhang, Ruoru Li, Sheng Chen, Shuhan Wu

**Affiliations:** 1 Shanghai Institute of Applied Physics, Chinese Academy of Sciences, Shanghai 201800, China; 2 Shanghai Advanced Research Institute, Chinese Academy of Sciences, Shanghai 201210, China; 3 University of Chinese Academy of Sciences, Beijing 100049, China

**Keywords:** dynamic imaging, coherent X-ray diffraction imaging, modulus enforced probe constraint, low frequency information transfer, static region constraints

## Abstract

Dynamic behavior is prevalent in biological and condensed matter systems at the nano- and mesoscopic scales. Typically, we capture images as “snapshots” to demonstrate the evolution of a system, and coherent X-ray diffraction imaging (CDI), as a lensless imaging technique, provides a nanoscale resolution, allowing us to clearly observe these microscopic phenomena. This paper presents a new dynamic CDI method based on zone-plate optics aiming to overcome the limitations of existing techniques in imaging fast dynamic processes by integrating the spatio-temporal dual constraint with a probe constraint. In this method, the modulus-enforced probe constraint and the temporal correlation of the dynamic sample low-frequency information are exploited and combined with an empty static region constraint in the dynamic sample. Using this method, we achieved a temporal resolution of 20 Hz and a spatial resolution of 13.2 nm, which were verified by visualized experimental results. Further comparisons showed that the reconstructed images were consistent with the ptychography reconstruction results, confirming the accuracy and feasibility of the method. This work is expected to provide a new tool for materials science and mesoscopic life sciences, promoting a deeper understanding of complex dynamic processes.

## 1. Introduction

The natural world abounds with microscopic dynamical behavior that defies direct visual observation. Modern imaging technologies (e.g., high-speed optical microscopy, electron microscopy, and coherent diffraction imaging) provide visual clues to reveal evolutionary mechanisms through the acquisition of time-resolved image sequences [1,2,3,4,5,6,7,8]. However, significant challenges remain in high-resolution nondestructive dynamic imaging. With the development of synchrotron radiation sources, significant improvements in the brightness and coherence of X-ray sources have greatly accelerated the development of X-ray microscopy and coherent X-ray diffraction imaging (CDI) technologies [9,10]. CDI, as a lens-less imaging technique that does not require an X-ray focusing element, has become a hotspot in high resolution imaging technology research in recent years due to its nanoscale resolution and non-destructive imaging capabilities. By recording the far-field diffraction pattern of the sample and using a phase retrieval algorithm, CDI can reconstruct the complex transmission function of the samples and achieve high-precision imaging. Its imaging resolution is primarily limited by the X-ray wavelength and the maximum diffraction angle (coherent photon flux), and can theoretically reach atomic-scale spatial resolutions.

Current CDI methods can be divided into two main categories: single-pattern CDI [11,12,13,14,15] and ptychography [16,17,18]. Compared to traditional single-pattern CDI, ptychography uses a scanning imaging scheme in which adjacent illuminated areas are partially overlapped, and exploits the overlap constraint to significantly improve the reconstruction quality and convergence speed, thereby demonstrating an extremely high resolution and robustness. This method has been extensively used for high-resolution imaging of static samples [19,20,21,22,23]. However, ptychography has inherent limitations when imaging dynamic samples [24,25,26,27,28], as fast changes in these samples disrupt the overlap consistency between scan positions, thereby degrading the reconstruction quality [29]. Although the development of multi-beam ptychography [30,31] and on-the-fly scans [32,33,34,35] facilitate the reduction of scan time, they still fall short of the needs in the research of rapidly changing dynamic samples. On the other hand, single-exposure ptychography [36,37], which uses a 2D grating to split the incident beam into multiple sub-beams, can provide overlapping regions without scanning. However, it limits the maximum diffraction angle of the exit wave, restricting the spatial resolution to the micron scale. Therefore, it is necessary to develop more efficient and robust single-pattern CDI techniques to achieve high-resolution imaging of dynamic sample processes.

Single-pattern CDI does not require a sample to be scanned; thus, it can potentially achieve very high imaging speeds and is particularly suitable for ultrafast dynamic imaging. However, the reconstructed image quality and reconstruction stability still have room for improvement. In recent years, many improved single-pattern CDI methods have been proposed to address the challenges of dynamic imaging. The improvements are mainly focused in two directions. The first is the introduction of a static region within the adjacent areas of the dynamic sample. This static region remains unchanged throughout the dynamic process, providing a robust real space constraint. However, adding a structured (spatially correlated CDI [38]) or independent (in situ CDI [39]) static region within the adjacent region of the sample greatly increases the complexity of the experimental sample preparation. In addition, this method typically uses plane wave illumination to achieve a larger illuminated area. However, due to the limited lateral coherence length of X-ray sources (about tens of microns), neither of these methods have been validated in X-ray CDI experiments. Coherent modulation imaging (CMI) is an alternative single-pattern CDI technique that is achievedby inserting a static structure (modulator) into the beam path [40]. The modulator effectively eliminates the inherent blur in CDI by breaking the Fourier transform relationship between the sample and the measured data. However, the accuracy of the modulator transfer function may somewhat compromise the optimal imaging resolution. Additionally, dynamic changes in the modulator, such as motion or vibrations, and beam jitter, can affect the image quality of dynamic CMI. The second direction considers the temporal correlation of the dynamic sample, which is manifested as temporal overlap (multiple-shot CDI) [41] or inter-frame similarity in the data set [42]. However, the constraint strength of this method typically weakens as the degree of change between adjacent frames increases. If there is no correlation between adjacent frames captured by the detector, this method fails.

Therefore, in this paper, we propose a new dynamic CDI (dynCDI) method with higher robustness based on Fresnel zone-plate (FZP) optics. This method can hopefully be applied to high-resolution imaging of various dynamically changing samples. In this method, under FZP probe illumination, by employing the modulus enforced probe (MEP) constraint [43] and the temporal correlation of the sample low-frequency information, together with the static region introduced in the dynamic sample as a real-space constraint, the entire dynamic sample image sequence and the unknown probe can be reconstructed quickly and stably. This “spatio-temporal dual constraint” dynCDI method is expected to significantly improve the stability and spatio-temporal resolution of dynamic sample imaging.

## 2. Methods

### 2.1. Static Region Constraint

**Figure 1:**
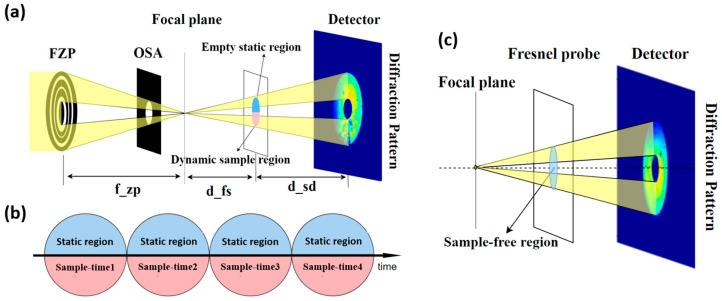
Schematic diagram of the light path in the dynCDI method. (**a**) Light path for CDI of dynamic sample. X-rays, passing through an FZP with a central stop and an order-sorted aperture, form a defocused probe on the sample and are ultimately detected by the downstream detector. (**b**) The time sequence of a dynamic sample, with the upside region selected as the static region while the downside sample changed over time under an external excitation. (**c**) The FZP probe illuminates a sample-free region on the silicon nitride film, and the generated diffraction pattern will be used in the MEP constraint in subsequent reconstructions.

The performance of CDI largely depends on the constraints during the imaging process. In single-pattern CDI, a support constraint is commonly used, while in ptychography, it appears as a spatial overlap constraint. The reconstructed image quality in single-pattern CDI is highly sensitive to the accuracy or compactness of the support, and the estimate of the support usually relies on prior knowledge, the sample autocorrelation function, or shrink-wrap algorithms. This process is complex and computationally intensive [44,45]. In the newly designed dynCDI method, an FZP probe is used (Figure 1a). This probe not only features a well-defined curvature wavefront, ensuring the uniqueness of phase retrieval [46], but also provides an excellent support for the sample reconstruction. By introducing a time-invariant static region adjacent to the dynamic sample, spatial overlap constraints are imposed on the reconstruction of dynamic sample time series, where the static region within the dynamic sample maintains consistency between different time frames, enhancing information redundancy and allowing simultaneous reconstruction of the unknown probe and the dynamic sample. In simulations, studies on the influence of various static region structures on the probe and sample reconstruction quality revealed that even an empty static region can result in a high reconstruction quality. As long as the sample preparation allows for relatively sparse regions, retaining an empty static region (Figure 1b) during the experiment is straightforward. At this point, the transmission function of the sample for each time frame is expressed as:

(1) $$O_{t}=\left(r_{1}\right)+S\left(r_{2}\right)$$

where $O_{t}\left(r\right)$ represents the sample transmission function that corresponds to the *t*-th diffraction pattern collected during the dynamic process of the sample. It will be abbreviated as the *t*-th frame transmission function in the following. *r* denotes the real-space coordinate. $D_{t}\left(r_{1}\right)$ is the dynamic region that varies over time, and $S\left(r_{2}\right)$ is the static region that remains unchanged over time.

### 2.2. Modulus Enforced Probe

The MEP technique was originally proposed in ptychography to minimize the crosstalk between a periodic sample and the probe during reconstruction [43]. We introduce the MEP constraint into our dynCDI method so that the crosstalk problem caused by the invariant static region can be addressed using the additional probe diffraction pattern. The MEP constraint not only provides a modulus constraint during reconstruction but also enables a probe pre-reconstruction using the hybrid input-output algorithm to obtain a good initial probe guess, thus speeding up the convergence of dynCDI. To ensure the accuracy of the experimental measurements, the MEP diffraction pattern is obtained by illuminating the silicon nitride (SiN) film area without any sample, considering that our samples are adsorbed on a SiN film. As shown in Figure 1c, the MEP diffraction pattern is captured after locating an empty area on the SiN film.

### 2.3. Transfer of Low-Frequency Information

Although the samples studied here exhibit dynamic changes, i.e., their structures at different time points may differ, their overall morphological profiles still remain similar during temporal evolution, especially for close time points. Therefore, in the reconstruction process, transferring Fourier low-frequency information between the sample transmission functions at adjacent time points helps significantly accelerate the iterative convergence of the entire dynamic sample image sequence. In the early stage (approximately the first 20 iterations out of the total 300 iterations) of image reconstruction where the convergence is unstable, we introduce the low-frequency information transfer operation into the iterative process, i.e., replacing the low-frequency components of the current frame transmission function with those from the last frame. The frequency range of the low-frequency transfer is determined by a pre-set parameter $q_{low}$ that is the maximum value of the spatial frequency to be transferred. The value of $q_{low}$ is related to the manner and extent of the sample variation, usually taking a few pixels in the reciprocal plane.

### 2.4. DynCDI Phase Retrieval Algorithm

**Figure 2:**
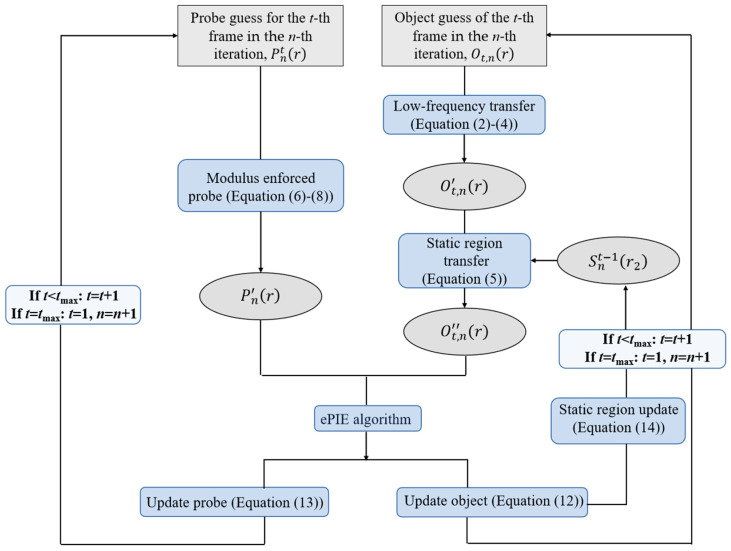
Flowchart of the new dynCDI algorithm. Initially, the guess functions for the sample and probe are input, then progressively updated using constraints such as MEP, low-frequency information transfer, and static region transfer. Finally, the ePIE algorithm is used to update the object and probe functions. The entire updating process is iterated repeatedly until a convergence criterion is met or the pre-set iteration number is reached. The interframe low-frequency transfer is typically used only in the first few iterations to speed up convergence.

Figure 2 shows a flowchart of the dynCDI algorithm we developed. First, the initial guess of the FZP probe P(r) and the initial guesses of the dynamic sample image sequence $O_{1}\left(r\right),O_{2}\left(r\right){,O}_{3}\left(r\right){,\cdots O}_{t}\left(r\right)$ are input to the algorithm. Then, the dynamic region $D_{t}\left(r_{1}\right)$ and the static region $S(r_{2})$ are determined by observing the approximate contour image of the sample in the holographic region of the diffraction pattern. Here, we define $O_{t,n}\left(r\right)$ as the transmission function of the *t*-th frame sample in the *n*-th iteration loop. If t ≠ 1, the relevant information from the transmission function update of the last frame sample can be used to guide the changes or updates of the current frame’s transmission function in the static region and low-frequency region of Fourier space.

The algorithmic update flow from the *t*-th frame to the (*t +* 1)th frame is as follows:

(1) Transfer the low-frequency information of the last frame transmission function to the current frame,

(2) $${Τ}_{t,n}\left(q\right)=F{[O}_{t,n}(r)];\;\;{Τ}_{t-1,n}\left(q\right)=F{[O}_{t-1,n}(r)]$$

(3) $${Τ}_{t,n}\left(q\right){|}_{{q\leq q}_{low}}={Τ}_{t-1,n}\left(q\right){|}_{{q\leq q}_{low}}$$

(4) $$O_{t,n}^{\prime }\left(r\right)=F^{-1}[{Τ}_{t,n}\left(q\right)]$$

where ${Τ}_{t,n}\left(q\right)$ is the Fourier transform of the object function at time *t*, i.e., ℱ${[O}_{t,n}(r)]$, representing the frequency distribution of the current frame sample transmission function. Its inverse Fourier transform yields a new current frame object function $O_{t,n}^{\prime }(r)$ after low-frequency transfer from the last frame object function.

(2) Transfer the static region information from the last frame object to the current frame to reflect the latest changes,

(5) $$O_{t,n}^{\prime }(r){|}_{r\in s}=S_{n}^{t-1}(r_{2})$$

where $O_{t,n}^{\prime }(r){|}_{r\in s}$ is the transmission function in the static region of the current frame object, while $S_{n}^{t-1}\left(r_{2}\right)$ is the static region transmission function that was synchronously updated when updating the previous frame object. The current frame object function updated by the static region transfer is denoted as $O_{t,n}^{\prime \prime }\left(r\right)$.

(3) Modify the probe with the MEP constraint. The probe function $P_{n}^{t}\left(r\right)$, updated in the previous frame updating during the *n*-th iteration (current iteration), is propagated to the detector plane, resulting in the diffraction wave amplitude distribution $A_{t,n}^{p}(k)$,

(6) $$A_{t,n}^{p}(k)=\left|F\left[P_{n}^{t}\left(r\right)\right]\right|=\left|P_{n}^{t}\left(k\right)\right|\;\;\;\;\;\;\;\;$$

The amplitude of the wavefront $P_{n}^{t}\left(k\right)$ is replaced by the square root of the experimentally measured probe diffraction intensity $I_{0}^{p}(k)$, resulting in a new wavefront denoted as:

(7) $$P_{n}^{\prime }\left(k\right)=P_{n}^{t}\left(k\right)\frac{\sqrt{I_{0}^{p}\left(k\right)}}{A_{t,n}^{p}(k)}$$

(8) $$P_{n}^{\prime }\left(r\right)=F^{-1}[P_{n}^{\prime }\left(k\right)]$$

After inversely Fourier-transforming the amplitude-corrected $P_{n}^{\prime }(k)$ and applying the support constraint, an updated probe function $P_{n}^{\prime }\left(r\right)$ is obtained.

(4) Now the exit wave $\psi_{t,n}$ of the *t*-th frame object in the *n*-th iteration is:

(9) $$\psi_{t,n}\left(r\right)=O_{t,n}^{\prime \prime }\left(r\right)\times P_{n}^{\prime }\left(r\right)$$

Its diffraction wave amplitude on the detector plane is:

(10) $$A_{t,n}\left(k\right)=\left|F\left[\psi_{t,n}\left(r\right)\right]\right|=\left|\Psi_{t,n}\left(k\right)\right|$$

The amplitude replacement is performed on the diffraction wavefront $\Psi_{t,n}(k)$ to obtain the modified wavefront:

(11) $$\Psi_{t,n}^{\prime }\left(k\right)=\Psi_{t,n}\left(k\right)\frac{\sqrt{I_{t}^{m}\left(k\right)}}{A_{t,n}\left(k\right)}$$

where $I_{t}^{m}(k)$ is the diffraction intensity of the *t*-th frame sample recorded by the detector. After applying the inverse Fourier transform to $\Psi_{t,n}^{\prime }(k)$, the modified exit wave $\psi_{t,n}^{\prime }(r)$ is obtained.

(5) Using the extended ptychographical iterative engine (ePIE) algorithm [47], the current frame sample function and the probe function are updated within the support range:

(12) $$O_{t,n+1}\left(r\right)=O_{t,n}^{\prime \prime }\left(r\right)+\alpha \frac{P_{n}^{\prime^{*}}\left(r\right)}{{\left|P_{n}^{\prime }\left(r\right)\right|}_{max}^{2}}\left[\psi_{t,n}^{\prime }\left(r\right)-\psi_{t,n}\left(r\right)\right]$$

(13) $$P_{n}^{t+1}\left(r\right)=P_{n}^{\prime }\left(r\right)+\beta \frac{{O_{t,n+1}}^{*}\left(r\right)}{{\left|O_{t,n+1}\right|}_{max}^{2}}\left[\psi_{t,n}^{\prime }\left(r\right)-\psi_{t,n}\left(r\right)\right]$$

where α and β are the relaxation parameters. Outside the support range, $O_{t,n+1}\left(r\right)$ = 1, $P_{n}^{t+1}\left(r\right)$ = 0.

(6) The static region of the updated current frame sample function is saved in $S\left(r_{2}\right)$ as the new static region transmission function:

(14) $$S_{n}^{t}\left(r_{2}\right)=O_{t,n+1}\left(r\right){|}_{r\in S}$$

After updating the object function, probe function, and static region function for the *t*-th frame sample, the above steps (1) to (6) are repeated for the (*t +* 1)th frame sample, until the last frame is updated, thus completing the *n*-th iteration and beginning the (*n +* 1)th iteration. This process is cycled until the transmission functions of all time-frame samples and the probe function converge, or the pre-set iteration number is reached.

## 3. Results

### 3.1. Simulation

**Figure 3:**
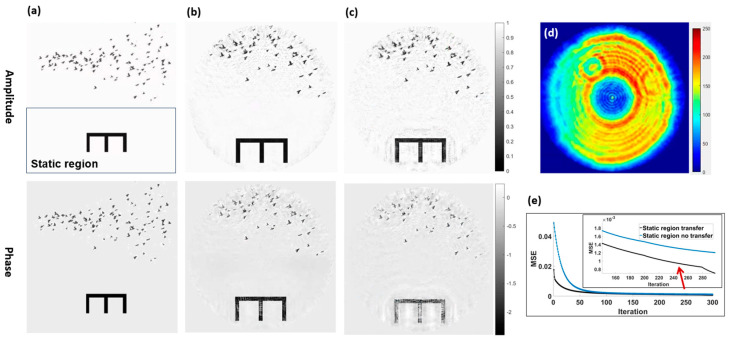
Simulative verification of the static region information transfer. (**a**) Original amplitude and phase of the 30th frame of the sample with a structured static region. (**b**) Reconstructed amplitude and phase with static region transfer. (**c**) Reconstructed results without static region transfer. (**d**) Probe amplitude reconstructed by ptychography in the validation experiment. This probe is also used as the incident probe in this simulation. (**e**) Convergence curves for both schemes.

In the simulation, we selected images of bird flock migration as the dynamic sample change process to be imaged, and designed a time-invariant region with a specific structure, referred to as the static region. The feasibility of the proposed method was verified by numerical simulations of CDI on the dynamic system. The total duration of the bird flock migration movie is 18 s. By uniformly selecting 49 frames from the migration movie, dynamic images of the sample at different time points were obtained. These images were then stitched with the designed static region, respectively, to create a complete time-series images of the dynamic sample. The static region, designed with a structure like the letter E, is located below the dynamic part of the sample (frame 30), as shown in Figure 3a.

The basic parameters of the FZP and the detector in the simulation were consistent with the experimental conditions of the BL08U1A beamline at Shanghai synchrotron radiation facility (SSRF) [48] for the actual experiments. The diameter of the FZP is 300 μm with a central stop of 80 μm and an outermost zone width of 30 nm. A defocused FZP probe with a diameter of 6 μm and photon energy of 705 eV was used to illuminate the sample. A set of dynamically evolving diffraction patterns were calculated by Fourier transforming the exit wave from each time frame of the sample, to simulate the data collected by the detector which was placed 105.4 mm downstream of the sample. The detector used is a sCMOS camera (Dhyana XV95, Tucsen Co., Ltd) with 2048 × 2048 pixels and a pixel size of 11 × 11 μm^2^. The probe (Figure 3d) used to generate the diffraction patterns is actually the probe reconstructed by ptychography in the verification experiment in Section 4. Random noise is added to each diffraction pattern to make the simulation results more realistic.

#### 3.1.1. Verification of Information Transfer in Static Region

To verify the effectiveness of static region information transfer in promoting reconstruction convergence, a static region constraint is introduced into the conventional CDI method to reconstruct the dynamic sample image sequence from simulated diffraction patterns. Figure 3b shows the amplitude and phase images of one frame of the dynamic object sequence reconstructed using the static region update and transfer technique. Figure 3c shows the reconstructed amplitude and phase images of the same sample frame using the conventional CDI method without static region transfer. It can be observed that when the static region image is transferred between adjacent frames, the artifacts in the dynamic image are significantly reduced and the convergence at the sample edges is more accurate. As shown by the mean-square error (MSE) convergence curves in Figure 3e, the convergence speed of image reconstruction is faster and the final error is smaller when the static region transfer constraint is used.

#### 3.1.2. Verification of the MEP Constraint and Low-Frequency Information Transfer

**Figure 4:**
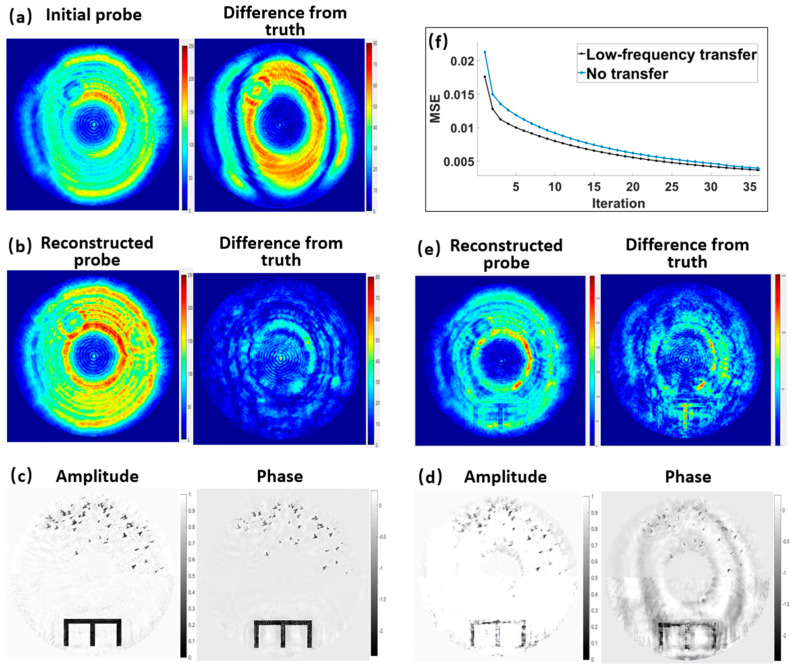
Simulative verification of the MEP constraint and low-frequency transfer in the new method. (**a**) Initial probe amplitude and its difference from the true probe. (**b**) Reconstructed probe amplitude using dynCDI with MEP and its difference from the true probe. (**c**) Reconstructed sample images of a frame using dynCDI with MEP. (**d**) Reconstructed sample images of the same frame without MEP. (**e**) Reconstructed probe amplitude without MEP and its difference from the true probe. (**f**) Comparison of convergence curves with and without low-frequency transfer.

In practical experiments, although the probe function can be reconstructed first by using the ptychography method, the probe spot during the dynCDI experiment may differ from that during the ptychography experiment. Therefore, in this section we discuss the ability of the dynCDI method to simultaneously reconstruct the probe and sample, as well as the role of the MEP constraint. Here, the initial probe was constructed by combining the ptychography-reconstructed probe (pty-probe) with the probe obtained by pre-reconstructing the MEP data [49]. The initial probe amplitude and its difference from the pty-probe are shown in Figure 4a. The probe reconstructed using the new dynCDI method and its difference in amplitude from the actual probe are shown in Figure 4b. Comparing Figure 4a,b, it is evident that the reconstructed probe is closer to the actual probe than the initial probe, indicating that the probe has been effectively recovered. Figure 4c shows the amplitude and phase images of a frame of the sample reconstructed by the new dynCDI method, where both the overall structure and the details of the sample are well restored. Figure 4d shows the amplitude and phase images of the same frame reconstructed without the MEP constraint, and Figure 4e shows the corresponding reconstructed probe and its amplitude difference from the actual probe. It can be seen that there is strong crosstalk between the sample and the probe. Therefore, the MEP technique is crucial in dynCDI.

The difference between a dynamic sample sequence and an individual sample group is that there is some similarity between dynamic sample structures in different time frames. Here, we verified the effectiveness of low-frequency information transfer between dynamic sample frames in improving CDI convergence. This technique helps to speed up the overall convergence of the dynamic image sequence. Figure 4f compares the convergence curves with and without low-frequency transfer, showing that low-frequency information transfer accelerates the convergence of the entire dynamic sequence during the early stage of reconstruction.

#### 3.1.3. Verification of Structureless Static Region Constraint

**Figure 5:**
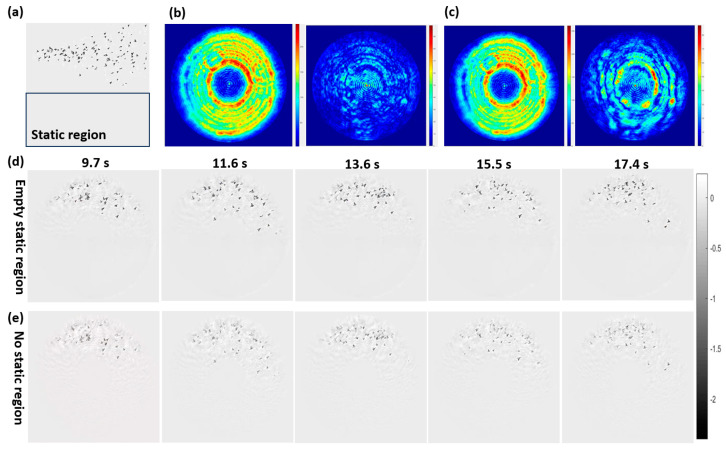
Simulative verification of the structureless static region constraint in dynamic sample imaging. (**a**) Schematic of the empty static region near the dynamic sample at 11.6 s. (**b**) Reconstructed probe amplitude under empty static region transfer and its difference in amplitude from the actual probe. (**c**) Reconstructed probe amplitude without static region transfer and its difference from the actual probe. (**d**) Reconstructed phase images of the dynamic sample with empty static region transfer. (**e**) Reconstructed images of the dynamic sample without static region transfer.

In experiments, it is very difficult to introduce a structured static region with spatial independence near the dynamic sample area. Therefore, a structureless empty region was proposed to be used as the static region, and we found that it can also improve the dynamic imaging performance in the simulative validation of this constraint. The simulation results are shown in Figure 5.

Figure 5a shows the structureless empty static region in the sample. The initial probe used for reconstruction is shown in Figure 4f. Figure 5b shows the reconstructed probe amplitude under the empty static region constraint and its difference in amplitude from the real probe (Figure 3d). It can be seen that this difference in amplitude is very small. Figure 5c, on the other hand, shows the reconstructed probe amplitude without static-region transfer and its difference in amplitude from the real probe. It can be seen that this difference in amplitude is significantly larger than in the empty static region case. Figure 5d shows the reconstruction results for the dynamic sample under the empty static region transfer condition. We can see that the overall sample structure as well as the edge details were correctly reconstructed. Figure 5e presents the reconstruction results of the dynamic sample without static region transfer. From Figure 5d,e, we can see that the reconstruction quality of the sample without the static region constraint is much worse than that with the empty static region constraint, with many details blurred and strong artifacts in the reconstruction results under no static region transfer.

### 3.2. Experiment

#### 3.2.1. Verification Experiment of DynCDI with a Moving Object

To verify the newly developed dynCDI method, we performed a soft X-ray dynCDI experiment at the BL08U1A beamline of SSRF. In the experiment, dynamic changes were achieved by moving the sample horizontally and ignoring the sample motion during detector exposure. This motion process was essentially part of the ptychographic scanning; thus, the ptychography reconstruction results can also be used for comparative validation.

**Figure 6:**
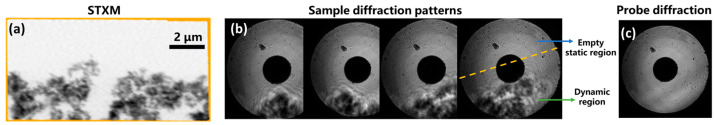
The images taken by the detectors in the experiment. (**a**) The STXM image of the sample with a size of 12 × 6 μm^2^. (**b**) The diffraction patterns of the sample collected by the far field detector. Due to the presence of a large holographic region in each diffraction pattern, the distribution contour of the sample can be seen and used to delineate the static and dynamic regions. Here, the area above the yellow dashed line is regarded as the static region, and the area below as the dynamic region. (**c**) The diffraction pattern of the FZP probe when an empty area on the sample is illuminated.

A suspension of Fe_3_O_4_ nanoparticles (100~200 nm) was dropped onto a SiN film to prepare for the extended sample. In the experiment, the X-ray photon energy was 705 eV, and a defocused probe of 6 μm diameter was generated by the FZP. The other parameters remained consistent with those in the simulation. Initially, a region-of-interest (ROI) on the sample was located using STXM imaging (Figure 6a). This ROI was specifically selected to have one side containing sample particles and the other side being empty, thereby creating spatially separated static and dynamic regions. Since the differences between transmission functions at different spatial positions on the SiN film are very small, even though the empty region is changing as the whole sample moves, we can still consider the transmission function of the empty region to be the same for each frame. Therefore, this empty region can be regarded as the static region for the whole time frame sequence.

During the experimental acquisition process of the dynCDI method, the sample was moved horizontally in steps of 0.3 μm, and the detector collected 40 diffraction patterns of the step-moving sample with an exposure time of 50 ms for each pattern (Figure 6b). We also captured one diffraction pattern for the probe illuminating a sample-free empty region (Figure 6c), which could be used for the MEP constraint. During the experimental data acquisition process of the ptychography method, we acquired 40 × 20 diffraction patterns using the same step size and exposure time.

**Figure 7:**
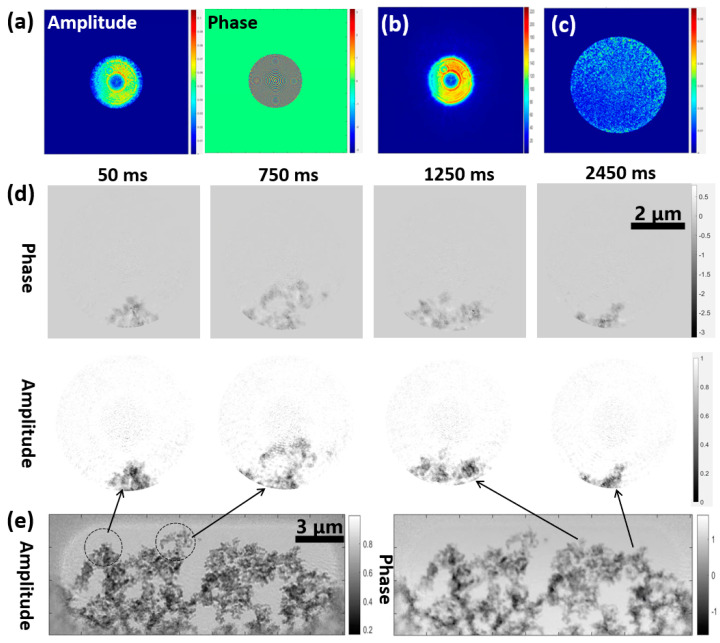
Reconstruction results for a moving sample using new dynCDI and ptychography with an FZP model probe as the initial probe. (**a**) Reconstructed probe amplitude and phase images using dynCDI. (**b**) Reconstructed probe amplitude by ptychography. (**c**) The difference in reconstructed probe amplitude between dynCDI and ptychography. (**d**) Dynamic sample phase and amplitude images reconstructed by dynCDI. Selected frames are shown. (**e**) Sample amplitude and phase images reconstructed by ptychography. The scan step size in the ptychography experiment is 0.3 μm with a scan grid of 40 × 20 points.

We used the proposed dynCDI algorithm to perform a 300-iteration reconstruction on 40 frames of the sample, obtaining the reconstructed probe image (Figure 7a) and reconstructed amplitude and phase results for the sample at different times (Figure 7d). To verify the accuracy of the reconstruction results, we further used the ptychography method to reconstruct the entire extended sample, obtaining another set of reconstructed probe (Figure 7b) and sample (Figure 7e) images. Figure 7c shows the amplitude difference in the reconstructed probe between the dynCDI method and the ptychography method. It can be seen that the probe reconstructed by the dynCDI method has some errors, with one reason for this being that the selected static region is a structureless SiN film, resulting in less diffraction encoding of the probe information in this region. In the MEP constraint, the probe also illuminates the structureless region, and the repetitiveness of the diffraction encoding may result in an insufficient sampling rate for the probe information in the empty static region, thereby decreasing the accuracy of the probe reconstruction. To solve this problem, the following methods can be used: the diffraction pattern of a structurally rich sample is also collected while the same probe is used to collect the diffraction patterns of the current dynamic sample, or time-invariant structures can be added in the static region during sample preparation to aid in probe reconstruction. These two methods require certain modifications to the experiment design, which are not discussed in this paper and can be considered as the research content for our future work.

**Figure 8:**
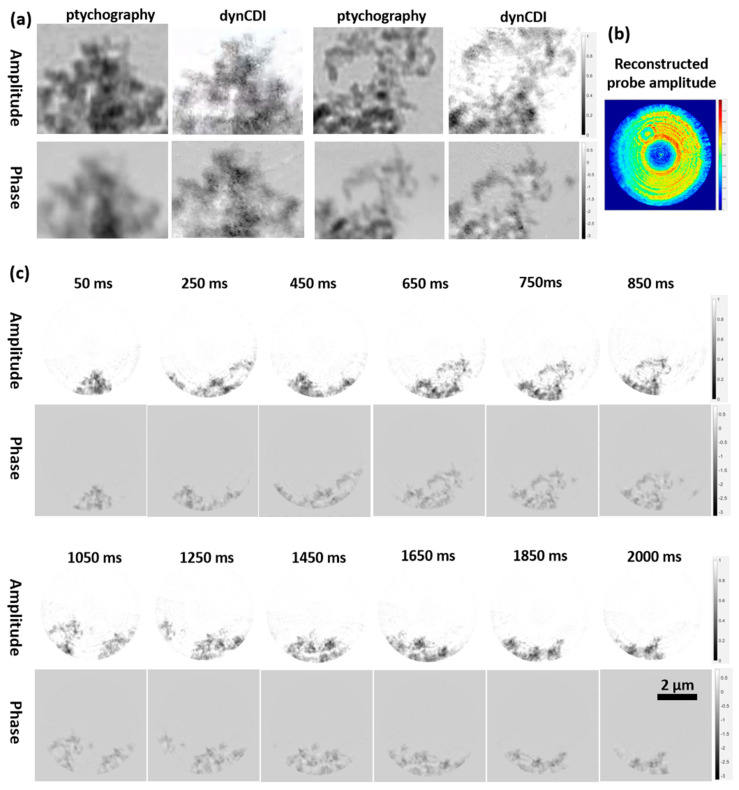
Reconstruction results of the new dynCDI method using the pty-probe as an initial probe guess. (**a**) The reconstruction results of ptychography and dynCDI are compared for two small regions selected from the sample. (**b**) The probe amplitude reconstructed by the dynCDI method. (**c**) The dynamic sample images reconstructed by dynCDI. Selected frames are shown here. Compared to the reconstruction quality in the guessed initial probe case (Figure 7d), it can be seen that using the pty-probe as the initial probe results in a cleaner image background and clearer sample edges in the dynCDI reconstruction. The video of the reconstructed images is provided in Supplementary Video S1.

The current experimental platform consists of a mature high-resolution ptychography imaging technology that can precisely reconstruct the probe. Therefore, the probe reconstructed by ptychography (pty-probe) can be used as the initial probe guess in dynCDI reconstruction. During the reconstruction process, only the sample functions were updated by the ePIE algorithm, while the probe update relied on the MEP constraint. The corresponding reconstruction results are shown in Figure 8. Figure 8a shows a comparison between ptychography-reconstructed images and dynCDI-reconstructed images (with the pty-probe as the initial guess) for two local areas marked by black circles in Figure 7e. Figure 8b shows the probe amplitude reconstructed by dynCDI. It can be seen that the dynCDI results are highly similar to the ptychography results both overall and in detail. Furthermore, the comparison of the reconstruction quality between the corresponding frames in Figure 7d and Figure 8c shows that using the pty-probe as the initial probe in the dynCDI reconstruction results in a better overall reconstruction quality for the dynamic image series (Figure 8c) with more details than the unknown probe case (using a guessed initial probe) (Figure 7d).

**Figure 9:**
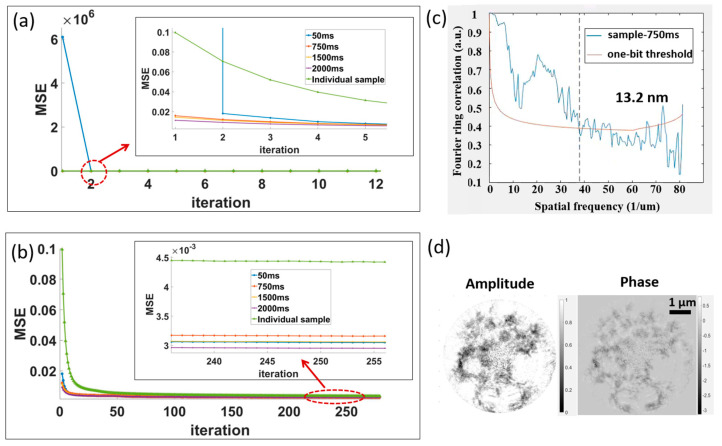
Convergence curves for the dynamic sample sequence and the independent sample reconstructed by the new dynCDI method. (**a**) Convergence curves for the initial iterations. (**b**) Complete convergence curves without the first iteration. (**c**) The spatial resolution estimate using FRC for the frame at 750 ms, obtaining a half-period resolution of 13.2 nm. (**d**) Reconstructed amplitude and phase of the independent sample.

To verify the effectiveness of interframe low-frequency information transfer and static region constraints in improving dynCDI imaging, we introduced a non-empty (no null region) sample as the 41st frame of the dynamic sequence during the dynamic sample reconstruction process. In the reconstruction, the sample does not share any information with the first 40 frames, but only uses the same probe. Therefore, the reconstruction results for the dynamic sample sequence and the independent non-empty sample (hereafter referred to as the independent sample) can be compared to demonstrate the effectiveness of the two constraints. Figure 9 shows the reconstruction error convergence curves of the dynamic sample and the independent sample.

Figure 9a compares the convergence curves of the dynamic sample at 50 ms, 750 ms, 1500 ms, and 2000 ms with that of the independent sample during the early iterations. It can be observed that, in the first iteration, the error of the first sample frame (50 ms) is relatively high, while the errors of the remaining frames are initially at a low level due to the transfer of low-frequency information and static region information from the previous frame and the increased number of MEP constraints. By looking at the inset in Figure 9a,b, it can be seen that the first frame of the sample with a smaller final error consistently has a higher error in the early iterations. This is due to the fewer transfer times of low-frequency and static region information to the first frame (the frame update sequence alternates, i.e., from frame 1 to frame 40, then back to frame 1). From the changes in the overall convergence curves, it can be seen that each frame of the dynamic sample with the low-frequency transfer and static region transfer converges faster than the independent sample, and each frame has a lower error after each iteration than the independent sample (except for the error of the first frame after the first iteration). This is mainly because the independent sample has no static region transfer and low-frequency transfer from others. The spatial resolution of a dynCDI-reconstructed image was estimated using the Fourier ring correlation (FRC) method (Figure 9c), resulting in a spatial resolution of 13.2 nm for the 750 ms frame. Figure 9d shows the reconstructed amplitude and phase images of the independent sample.

#### 3.2.2. Application Experiment: Stretching a Carbon Fiber Film

**Figure 10:**
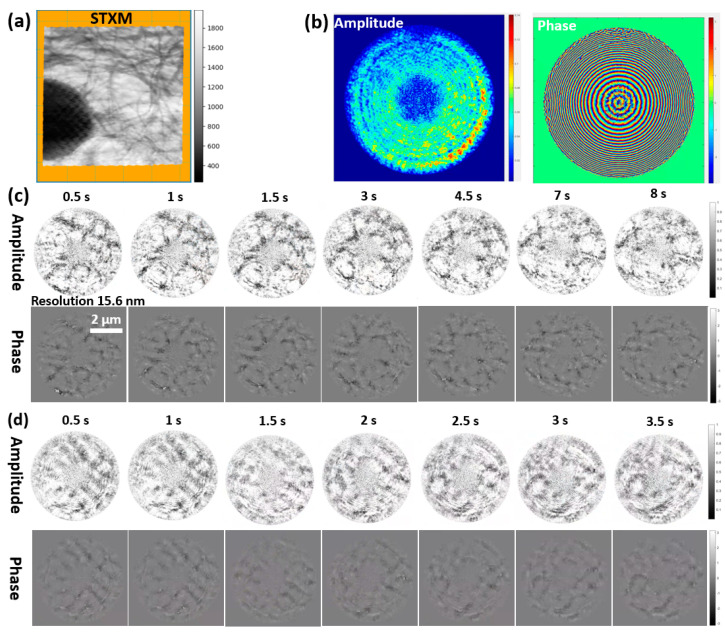
Reconstruction results of the carbon fiber stretching experiment. (**a**) STXM image of the carbon fiber film with a size of 10 × 10 μm^2^. (**b**) Reconstructed probe amplitude and phase using the dynCDI method. (**c**) Reconstructed images of the sample before breaking (first data set), totaling 20 frames. The resolution of the frame at 0.5 s is estimated to be 15.6 nm using the FRC method. (**d**) Reconstructed sample images after breaking (second data set), totaling 20 frames. Videos of the two sets of reconstruction results are available in Supplementary Videos S2 and S3.

In this experiment, a carbon fiber film of a certain thickness was mounted on a stepper motor to achieve film stretching by motor movement. The parameters for the illumination probe used were the same as in the above validation experiment. The exposure time was set to 50 ms and the detector acquisition interval was 500 ms, with the motor moving 0.01 mm per step. Sample motion was ignored during exposure (although in reality there may be slight vibrations). This experiment did not set up spatially separated static and dynamic regions, and only low-frequency information transfer and MEP constraints were considered in the reconstruction. Figure 10a shows the absorption contrast of the film measured by STXM. Figure 10b displays the reconstructed probe amplitude and phase after 300 iterations of the dynCDI method, where the initial probe was a theoretical FZP model probe. Figure 10c shows reconstructed images of the film from the first set of measured data (early stage). Figure 10d shows the reconstructed images at another position of the film from the second set of measured data (later stage). In the first set of reconstruction results, the unbroken carbon fiber film showed some elasticity during the motor movement, causing the initial frames to change more slowly compared to the second set of reconstructed images where breakage had occurred. The subsequent relaxation changes in the first set were more pronounced and lasted longer.

#### 3.2.3. Application Experiment: Heating Fe_3_O_4_ Nanoparticles Attached to a Copper Grid

**Figure 11:**
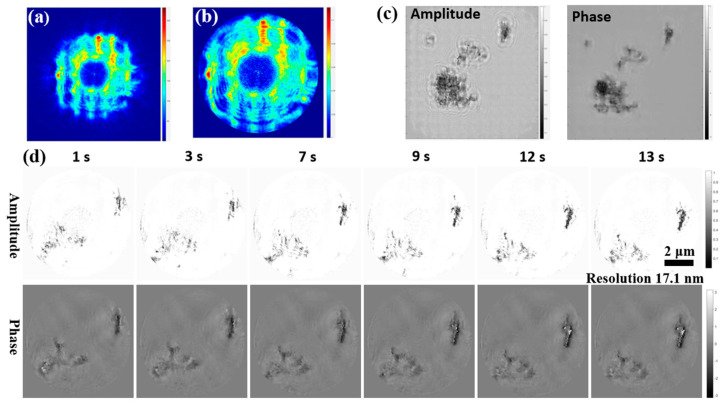
Reconstruction results of the nanoparticle heating experiment. (**a**) Probe amplitude reconstructed by ptychography. (**b**) Probe amplitude reconstructed by the new dynCDI method (using the pty-probe as the initial probe). (**c**) Reconstructed sample amplitude and phase by ptychography. (**d**) Reconstructed sample images obtained by the new dynCDI method at different time points during heating. The resolution of the sample image at 13 s is estimated to be 17.1 nm using the FRC method. A video of the reconstructed images is provided in Supplementary Video S4.

In this experiment, the heating stage was installed inside the experimental chamber, and the heated sample was positioned in the beam path. The sample was then heated by heating the sample holder. A defocused FZP probe with an X-ray energy of 645 eV and a diameter of 7 μm was used in the experiment. The sample status was measured as the temperature increased from 50 °C to 60 °C. The detector exposure time was set to 80 ms, and the data acquisition interval was set to 1 s, ignoring sample changes during exposure. A total of 120 frames of data were collected, but only 13 frames were selected for dynCDI reconstruction due to the slow sample change. A ptychography reconstruction was also performed for the sample at the starting temperature. Figure 11a,c show the probe and sample images reconstructed by ptychography. Figure 11b shows the reconstructed probe amplitude using the new dynCDI method with the pty-probe as the initial probe guess. The reconstructed dynamic sample images obtained by the new dynCDI method are shown in Figure 11d. It can be observed that during the heating process, the sample drifts towards the side with a lower temperature (during cooling, the drift direction is opposite). This phenomenon is caused by the expansion of the sample holder with increasing temperature [50]. In this experiment, the FZP probe appeared unstable during imaging, which may be due to heat conduction from the heating stage to the FZP stage, resulting in poorer reconstructed probe images.

## 4. Discussion

The existence of the hollow area in the center of the FZP probe has a significant negative impact on the imaging quality of this method, and brings disturbances to the delineation of the static and dynamic regions as well as to the determination of the support. However, the FZP probe pattern can be measured directly with the detector, thus providing diffraction data for the MEP constraint; in addition, the high curvature of the FZP probe wavefront is favorable for the fast convergence of the dynCDI method. In the future, the method will be extended to probe cases without a central hole, such as pinhole probes and KB mirror-focused probes. Furthermore, a structured probe with a high degree of phase diversity, such as the spot produced by a phase modulator, may be a more desirable dynamic CDI probe that has no central hole but a phase distribution with a high rate of change, and thus is one of the directions for the development of the dynCDI method.

The proposed dynCDI method involves two fundamental assumptions: (1) The FZP probe is assumed to remain static throughout the diffraction data acquisition process. The validity of this assumption depends on the stability of the incident X-ray beam during dynamic imaging. Under normal synchrotron experimental conditions, the data acquisition time for dynamic sample imaging is typically so short that the probe can be considered sufficiently stable. This assumption generally does not affect the reconstruction accuracy. (2) Motion or change in the sample can be ignored during the detector exposure which is equivalent to assuming that the detector exposure time is very short compared to the sample change rate. This assumption is generally independent of the sample, but depends on the detector acquisition speed (here 24 fps). The need to acquire diffraction signals of sufficient strength requires that the exposure time remain above a certain level; this requirement can be mitigated by increasing the incident beam intensity. Consequently, the temporal resolution of dynamic process imaging is fundamentally limited by the detector acquisition rate. Therefore, advances in detector technology with increased frame rates would allow the study of faster dynamic phenomena.

## 5. Conclusions

In summary, this paper presents a new dynCDI method suitable for imaging dynamic processes with different change rates. In this method, an FZP probe is used to illuminate a dynamic sample with a structureless static region, which is combined with the MEP technique and the exploitation of low-frequency information similarities between sample structures at adjacent moments, to achieve dynamic imaging with high spatiotemporal resolution. We verified the feasibility of this method through computer simulations and demonstrated it in synchrotron radiation experiments. In the study, the feasibility of experimental implementation was fully considered, and the effectiveness and easy operation of constructing the empty static region in the ROI of the sample were proposed and verified. In validation experiments, the sample structures reconstructed by the new dynCDI method were highly consistent with the ptychography reconstruction results, and we achieved a temporal resolution of 20 Hz and a spatial resolution of 13.2 nm with the new method. These results represent significant improvements over that of multi-shot CDI [41] (8 keV, 20 Hz, 158 nm) and PID3Net-based single-shot CDI [51] (5 keV, 1 Hz, 40 nm). We also applied this method to experimental observations of carbon fiber film stretching and Fe_3_O_4_ nanoparticle heating. The above simulation and experimental results fully verify the effectiveness of the developed dynCDI method.

In particular, this dynamic imaging capability has broad application prospects in advanced materials characterization. For instance, the dynCDI method enables real-time observation of material structure evolution in lithium-ion battery electrodes during charge–discharge cycles [39], as well as the co-deposition behavior of nanomaterials under applied voltage conditions [24]. Beyond materials science, the dynCDI method is of equal importance in life sciences by providing pivotal technical support for live-cell dynamic imaging [52]. In the practical implementation of the method, researchers only need to construct an appropriate in situ sample stage (equipped with external stimulation devices: electrical, thermal, mechanical, or magnetic) tailored to fit the investigated object, then synchronize the signal acquisition with the stimulus activation, and finally reconstruct the dynamic process image series through the dynCDI algorithm. This method is expected to promote the application and advancement of CDI technology in the research of complex dynamic processes.

## Data Availability

Data will be made available upon reasonable request.

## Supplementary Materials

The following supporting information can be downloaded at: https://www.mdpi.com/article/10.3390/s25072323/s1, Video S1: Animation of reconstructed images of a moving sample in the verification experiment, Video S2: Animation of reconstructed images before fracture in the carbon fiber film stretching experiment, Video S3: Animation of reconstructed images after fracture in the carbon fiber film stretching experiment, Video S4: Animation of reconstructed images of nanoparticles in the heating experiment.
